# TRPs in Ovarian Serous Cystadenocarcinoma: The Expression Patterns, Prognostic Roles, and Potential Therapeutic Targets

**DOI:** 10.3389/fmolb.2022.915409

**Published:** 2022-06-24

**Authors:** Cheng Zhang, Cong Xu, Chuanshun Ma, Qinghua Zhang, Siyuan Bu, Dao-Lai Zhang, Liting Yu, Hongmei Wang

**Affiliations:** ^1^ Department of Protein and Antibody Engineering, School of Pharmacy, Binzhou Medical University, Yantai, China; ^2^ Department of Pharmacology, School of Medicine, Southeast University, Dingjiaqiao 87, Nanjing, China

**Keywords:** ovarian cancer (OV), transient receptor potential (TRP) channel, biomarker, therapeutic target, bioinformatic

## Abstract

Ovarian cancer (usually ovarian serous cystadenocarcinoma, or OV) is the fifth leading cause of cancer-related deaths in women, with more than 184,000 deaths reported worldwide annually, and is a highly malignant carcinoma. However, the mechanism of etiology remains unclear. The lack of prognostic and diagnostic biomarkers is a main limitation for clinical diagnosis and treatment. The transient receptor potential (TRP) channels play essential roles in the occurrence and development of cancers which may have the potential as a therapeutic target for OV. In our study, we used bioinformatic methods to study the potential effect and function of the TRP family in patients with OV. Differential expression analysis showed that the expression of TRPC7, TRPV4, and other TRP family members was significantly different between tumor and normal tissues. Through survival analysis, we screened out that the high expression of TRPC7, TRPV4, and TRPM (2,4,8) was negatively correlated with the prognosis of patients. In contrast, the low expression of TRPM3 was negatively associated with the prognosis. Cox regression analysis further indicated that TRPV4 was OV’s most likely therapeutic target. Finally, we conducted mRNA expression analysis, functional enrichment analysis, and immune infiltration analysis to confirm that TRPV4 was the most convincing therapeutic target of OV.

## Introduction

Ovarian cancer accounts for about 3% of cancers in women (Stewart et al., 2019). The most common histological subtype of ovarian cancer is ovarian serous cystadenocarcinoma (OV), accounting for about 90% of all ovarian cancers (Liu et al., 2020; Shi et al., 2021). In 80% of women, the disease is usually advanced once diagnosed, with a low five-year survival rate and a poor prognosis. The incidence of ovarian cancer fell by less than one percent in the last 2 decades, but the death rate remained unchanged (Stewart et al., 2019). Moreover, morbidity is on the rise in middle-income countries (Webb and Jordan, 2017). The detection of ovarian cancer at an earlier stage mainly relies on combining annual CA-125 tests with ultrasound imaging. The latest research suggested that developing targeted therapies for ovarian cancer patients based on understanding the biological pathways and targets involved in the development of the cancer process would provide excellent help for ovarian cancer diagnosis and treatment. For the lack of remarkable therapeutic targets and predictive biomarkers, the standard treatment for ovarian cancer is still surgery followed by combination chemotherapy (Stewart et al., 2019). Molecularly targeted therapies are revolutionized therapeutics, which interfere with specific molecules to block cancer growth, progression, and metastasis (Lee et al., 2018). Understanding the molecular and genetic changes in ovarian cancer leads to the development of targeted research on ovarian cancer (Grunewald and Ledermann, 2017). At present, several biomarkers of ovarian cancer have been found, such as *COL11A1* and *POSTN* (Wu et al., 2014; Wu et al., 2021; Yue et al., 2021). *COL11A1* mediates the upregulation of *p-Sp1* and finally induces the increased release of TGF-β3 and interleukin-6, thus promoting the proliferation and migration of ovarian cancer cells (Wu et al., 2021). *POSTN* is highly enriched in fibroblasts, and its overexpression is related to the decrease in overall survival (OS) rate. *POSTN* can activate *PI3K/AKT* pathway to promote the proliferation and migration of ovarian cancer cells (Yue et al., 2021).

TRP non-selective cation channels constitute a superfamily (Pan et al., 2011). These channels respond to multiple types of stimuli and, in turn, allow cation entry (Moran, 2018). Recent studies have shown that TRP channels mediated intracellular Ca^2+^ regulation, and mutations in the TRP genes affected the temporal and spatial distribution of Ca^2+^, resulting in the dysregulation of various downstream effectors. In addition, TRPs mutation is also related to the pathophysiological features of cancers, including the enhanced ability of cancer cells to proliferate, survive and invade (Yang and Kim, 2020). The TRP superfamily comprises 28 cation osmotic channels in mammals, divided into six subfamilies based on the sequence homology of amino acids (Pan et al., 2011). The first studied subfamily of mammalian TRP channels is the TRPC subfamily or standard TRP channel. Twenty-five years ago, the first mammalian TRPC channel was cloned, opening the vast horizon of the TRPC field (Chen et al., 2020). TRPC1 is a non-selective cation channel for Ca^2+^ and Na^+^ and is associated with cancer cells adhesion and drug resistance. The upregulation of TRPC1 expression in breast cancer tissues and cells is associated with breast cancer metastasis (Zhang et al., 2020). TRPC3 expression varies between tissue and cell phenotypes in different developmental states (Tiapko and Groschner, 2018). TRPC3 plays a significant role in proliferating cells, vascular progenitor cells (Poteser et al., 2008; Hao et al., 2018), and differentiated cells (Li et al., 1999). TRPV channels can regulate the proliferation, apoptosis, angiogenesis, migration, and invasion of cancer cells during tumor progression. Up and down-regulation of TRPV expression have been reported depending on different stages of cancer; this may be due to the increased expression of active TRPV channels in the plasma membrane would contribute to the Ca^2+^-dependent proliferation response of cancer cells (Santoni et al., 2011). TRPV2 is regarded as a therapeutic target of leukemia for its inhibition directly leads to the apoptosis of cancer cells (Siveen et al., 2020). The TRPM family is the largest and most diverse subfamily of the TRP superfamily (Huang et al., 2020). TRPM4 has been extensively studied in the prostate and is over-expressed in benign and malignant prostate tissues. Using cDNA microarray technology, transcription of the TRPM4 gene was first associated with the malignant transformation of prostate intraepithelial neoplasia to invasive prostate cancer in 2004 (Gao and Liao, 2019). These findings suggested that TRP channels worked on cells in various ways and played a key role in cancer progression. TRP channels are also promising targets for drug discovery (Kaneko and Szallasi, 2014).

Many studies have been carried out on targeted therapy for OV, but it is still unclear whether TRPs can be regarded as a biomarker and therapeutic target for OV. In this study, we analyzed the TRP family by bioinformatics and finally found that the high expression of *TRPV4* may be involved in the occurrence and poor prognosis of OV, and it could be used as the therapeutic target for OV. We hope to provide potential biomarkers for targeted therapy of OV through this research.

## Materials and Methods

### Data Acquisition

TCGA (https://www.cancer.gov/about-nci/organization/ccg/research/structural-genomics/tcga) is a publicly available database covering more than 10,000 patient samples from various human cancer types, including clinical pathology and bioinformatic data (Hutter and Zenklusen, 2018). The clinical features of patients obtained from the TCGA database included gender, race, age, and pathological stage. A lot of information about OV patients used in this study came from this database.

### Differential Expression Analysis

The RNA-Seq data of OV patients were downloaded from the TCGA database. The differential expression analysis was performed using R software for the Wilcoxon test, and a box diagram visualized the results.

### Pathological Staging Analysis

GEPIA (http://gepia.cancer-pku.cn/index.html) is a web server for gene expression profiling and interactive analyses in cancer and normal tissues (Tang et al., 2017). We chose the “stage plots,” on this website and used the OV dataset to analyze the differential expression of the TRP family among pathological stages.

### Kaplan–Meier Survival Analysis

The Kaplan–Meier plotter (https://kmplot.com/analysis/) is capable of assessing the effect of 54k genes (mRNA, miRNA, and protein) on survival in 21 cancer types including breast (*n* = 7,830), ovarian (*n* = 2,190), lung (*n* = 3,452), and gastric (*n* = 1,440) cancer. Sources for the databases include GEO, EGA, and TCGA. We selected the OV database on this website, input the candidate genes, and chose OS (RFS) to perform survival analysis. A statistically significant difference was considered when the *p*-value was less than 0.05.

### Construction of Prognostic Risk Score Model

To determine the prognostic factors of OV by multivariate Cox analysis, we constructed a risk score (RS) model. The RS was calculated as follows: $\mathrm{RS}=\sum_{i=1}^{n}\left(Xi*Yi\right)$ (X: coefficients; Y: expression level of genes). Then, we figured the RS of each OV patient and divided all patients into high-risk and low-risk groups, with the median RS as the cut-off value. To evaluate the discrimination and predition ability of RS models, we used the Survival and SurvivalROC packages of R software to plot Kaplan-Meier (K-M) curves and receiver operating characteristic (ROC) curves and calculated the area under the curve (AUC). The R software version V4.0.3 (R Foundation for Statistical Computing, 2020) was used for the current analysis.

### Gene Alteration Analysis

cBioPortal (www.cbioportal.org) is a comprehensive open network platform based on the TCGA database, integrating data mining, data integration, and visualization (Cerami et al., 2012). On this website, we chose the OV dataset to study the genetic alterations of the TRP family.

### Gene Function Prediction

GeneMANIA (http://www.genemania.org) is used to predict the function of related genes and look for genes with similar functions. In this study, the functions of the TRP family were predicted using this database.

### GO Enrichment and KEGG Pathway Analysis

DAVID (https://david.ncifcrf.gov) bioinformatic resources consist of an integrated biological knowledgebase and analytic tools to systematically extract biological meaning from large gene/protein lists (Huang et al., 2009a; Huang et al., 2009b). In this study, we performed the Gene Ontology (GO) and Kyoto Encyclopedia of Genes and Genomes (KEGG) pathway enrichment analysis on the TRP family. TRP family’s function and pathway terms were visualized using a mapping website called “bioinformatics (http://www.bioinformatics.com.cn/)”.

### Immune Correlation Analysis

The TIMER (https://cistrome.shinyapps.io/timer/) web server is a comprehensive resource for systematical analysis of immune infiltrates across diverse cancer types (Li et al., 2016a; Li et al., 2017). We input the TRP genes and selected the OV dataset to analyze the relationship between the TRP family and immune cell infiltration.

### Gene Correlation Analysis

UALCAN (http://ualcan.path.uab.edu/analysis.html) is an easy-to-use interactive portal for in-depth analysis of TCGA gene expression data (Chandrashekar et al., 2017). In this study, we entered “TRPV4” and selected the OV dataset to analyze genes related to TRPV4.

## Results

### Expression Levels of TRPs in OV

**FIGURE 1:**
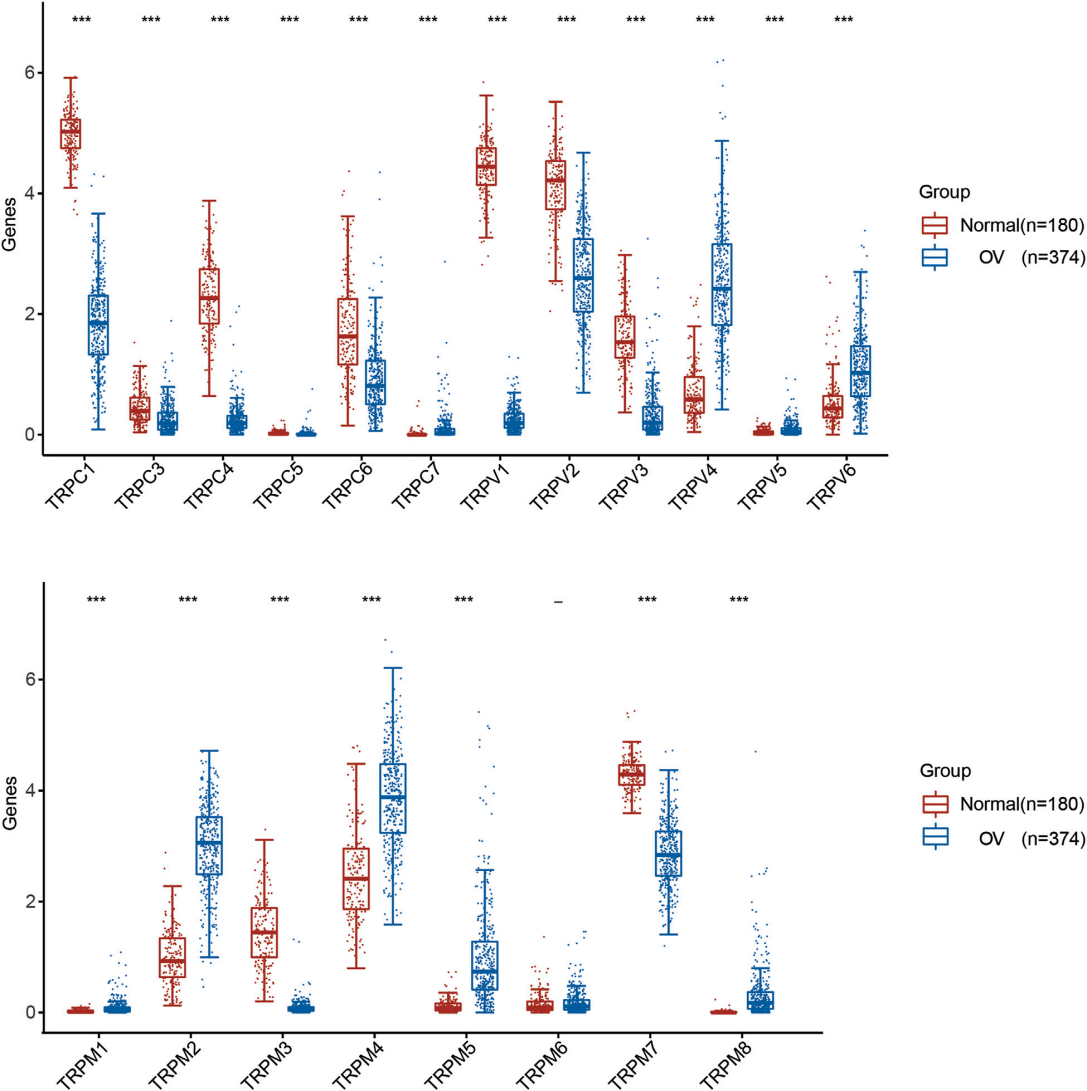
Differential expression of TRP family in OV. Annotation: Box plot showed the differential expression of TRPs between cancer tissues and adjacent normal tissues in OV of TCGA database. Blue represented the expression level in cancer tissues, and red represented the expression level in normal tissues.

**FIGURE 2:**
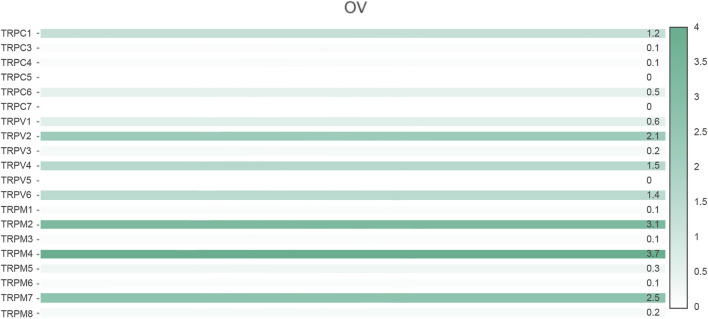
Relative expression of TRP family in OV (GEPIA). Annotation: Histogram showed the relative expression of TRPs in OV. The depth of color represented the level of relative expression and the numbers marked on the histogram represented the specific values of relative expression.

**FIGURE 3:**
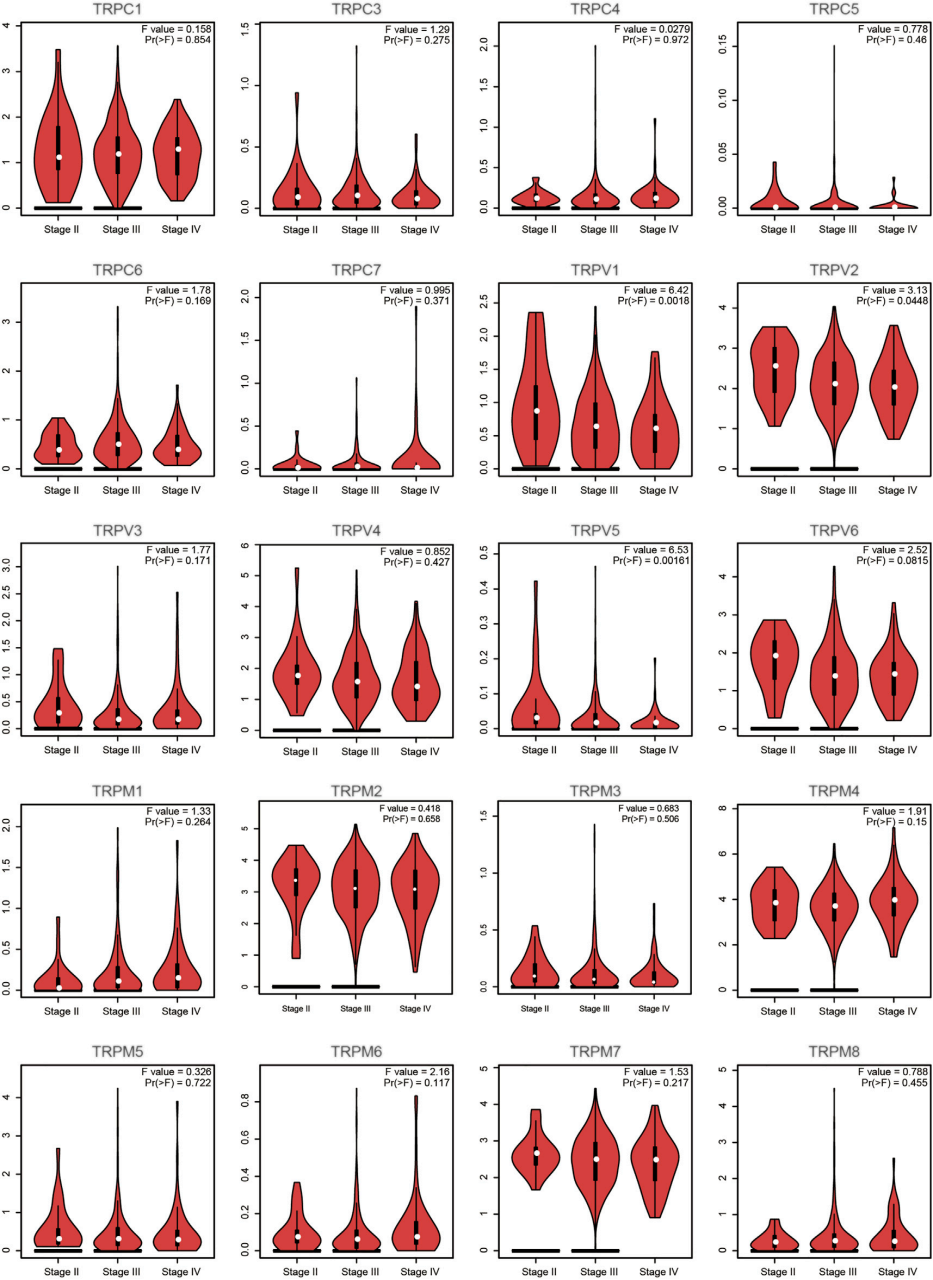
Correlation between TRP family and pathological stages of OV patients (GEPIA). Annotation: Violin plot showed the correlation between TRPs and pathological stages. The *p* values between different stages were shown for each TRP gene. A statistically significant difference was considered when the *p*-value was less than 0.05.

First, we compared the expression of TRP genes between cancer and normal groups. The results showed that the expression levels of TRPC7, TRPV (4,5,6), and TRPM (2,4,5,8) in the OV group were significantly higher than those in the normal group. In comparison, the expression levels of TRPC (1,3,4,6), TRPV (1,2,3), and TRPM (3,7) in the OV group were significantly lower than those in the normal group (Figure 1). We also compared the relative expression levels of the TRP family in OV and found that among these TRP genes, the relative expression levels of TRPV2 (2.1), TRPV4 (1.5), TRPM2 (3.1), TRPM4 (3.7), and TRPM7 (2.5) were higher (Figure 2). Then, we evaluated the correlation between the expression of TRPs and the pathological stages of OV patients and found that the expression levels of TRPV1 (*p* = 0.0018), TRPV2 (*p* = 0.0448), and TRPV5 (*p* = 0.00161) were significantly correlated with the pathological stages of OV patients (Figure 3). Taken together, the expression levels of TRPV4, TRPM2, and TRPM4 in OV tissues were significantly higher than those in the adjacent normal tissues, and they were also relatively higher among all the TRP genes. Therefore, TRPV4, TRPM2, and TRPM4 may play an important role in the occurrence and development of OV.

### Prognostic Value of Individual TRP in OV Patients

**FIGURE 4:**
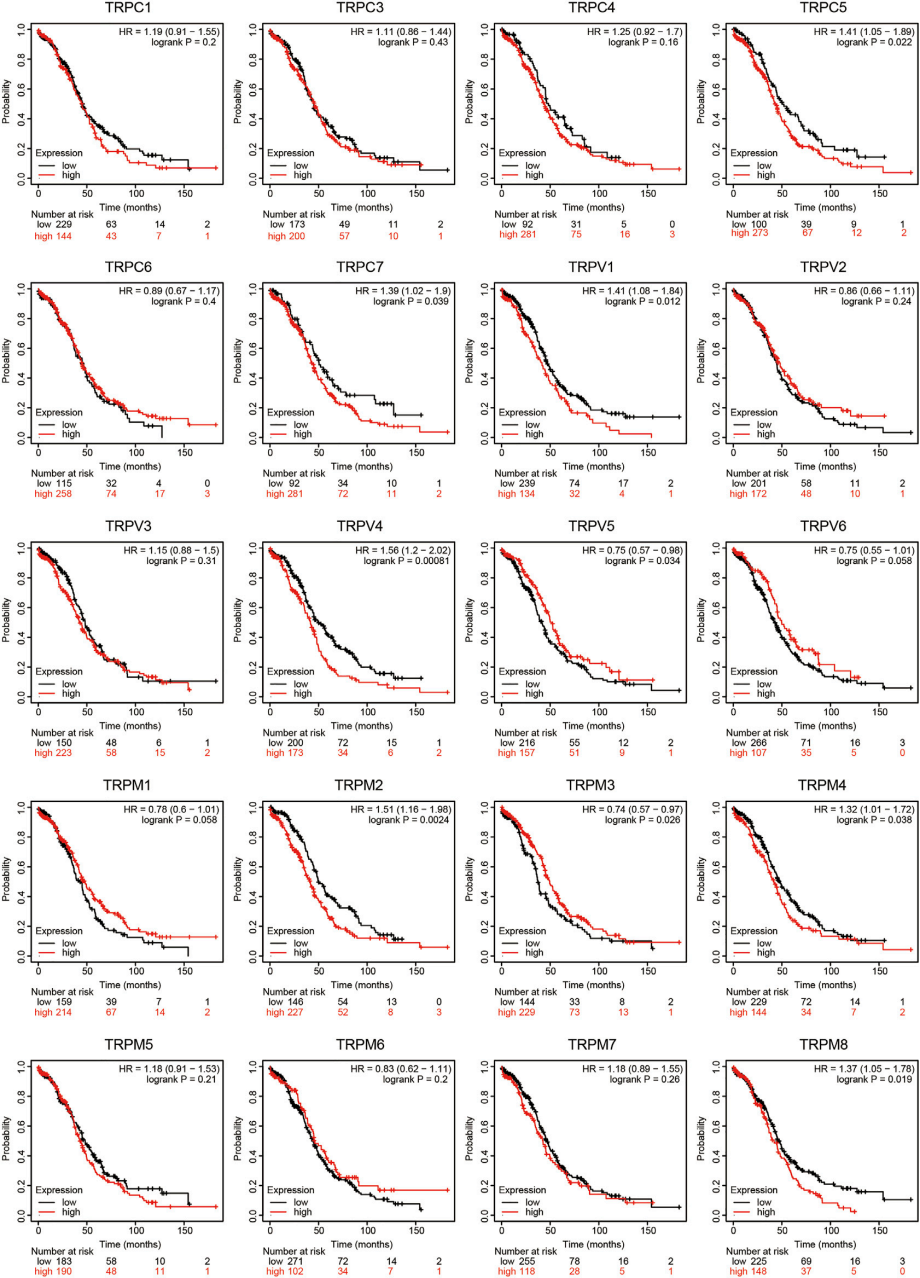
OS curves of TRPs expression in OV patients (Kaplan-Meier plotter). Annotation: The OS curves were performed using K-M Plotter database, with representations of different samples from low (black lines) and high (red lines) TRP expression group, respectively. + denoted censored observations. The log-rank test was used to compare the OS between groups, *p* < 0.05 was considered statistically significant.

**FIGURE 5:**
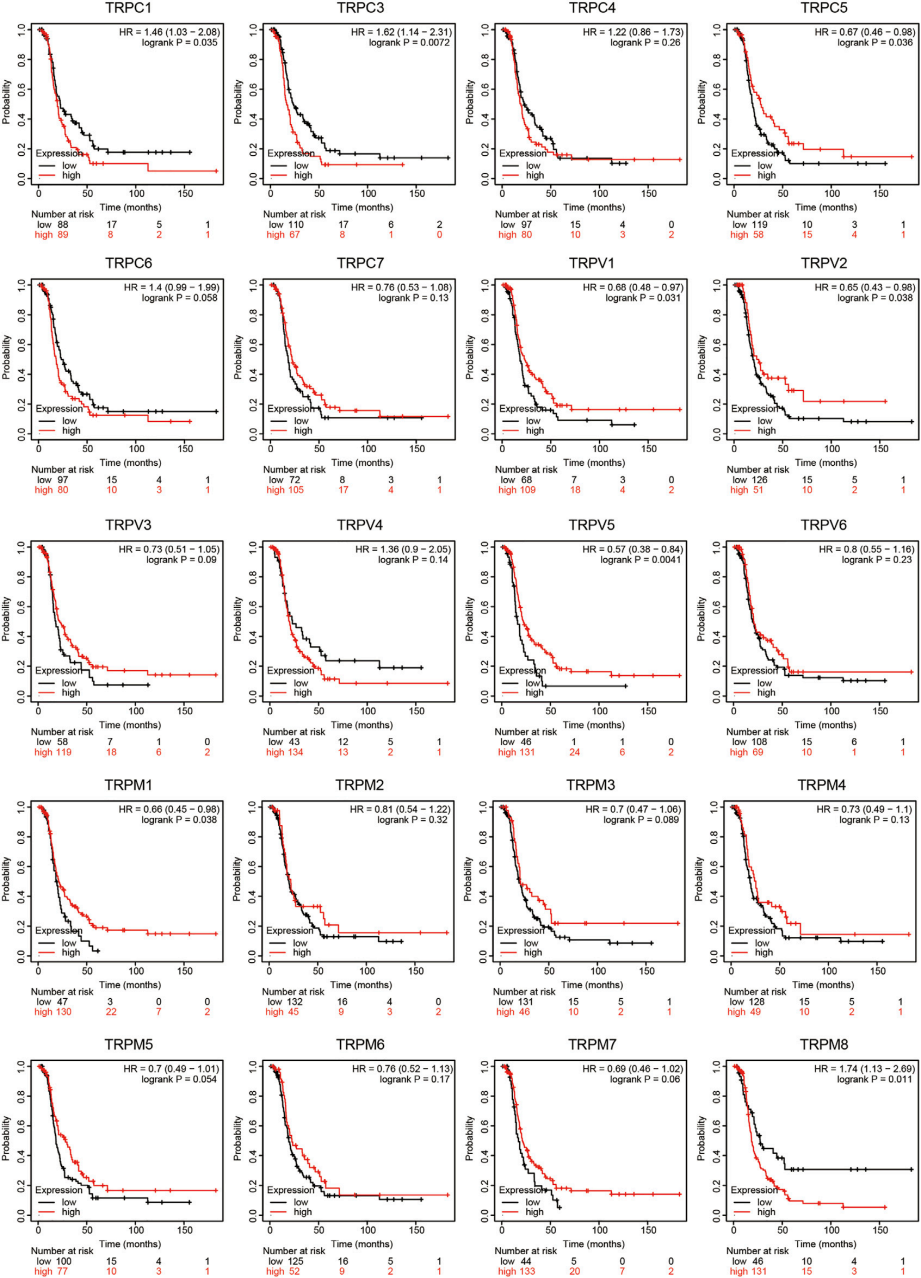
RFS curves of TRPs expression in OV patients (Kaplan-Meier plotter). Annotation: The RFS curves were performed using K-M Plotter database, with representations of different samples from low (black lines) and high (red lines) TRP expression group, respectively. The log-rank test was used to compare the RFS between groups, *p* < 0.05 was considered statistically significant.

To evaluate the prognostic value of the TRP family for OV patients, the Kaplan-Meier plotter was used to conduct a K-M survival analysis. The OS curves are shown in Figure 4. Among them, TRPC5, TRPC7, TRPV1, TRPV4, TRPV5, TRPM2, TRPM3, TRPM4, and TRPM8 were significantly correlated with OS of OV. The high expression of TRPC5, TRPC7, TRPV1, TRPV4, TRPM2, TRPM4, and TRPM8 indicated a shorter OS period, while lower expression levels of TRPV5 and TRPM3 showed poorer prognosis in OV. Furthermore, the recurrence-free survival (RFS) curves in Figure 5 showed that TRPC (1,3,5), TRPV (1,2,5), and TRPM (1,8) were significantly correlated with RFS of OV, in which the high expression of TRPC1, TRPC3, and TRPM8 suggested short RFS, and the low expression of TRPC5, TRPV1, TRPV2, TRPV5, and TRPM1 suggested short RFS. Combined considering the expression levels of TRP genes in the OV, we concluded that TRPC7, TRPV4, and TRPM (2, 4, 8) were up-regulated in OV and negatively related to the OS of patients. The TRPM3 was down-regulated in OV and showed a shorter OS in patients.

### Univariate and Multivariate Cox Regression Analysis

**FIGURE 6:**
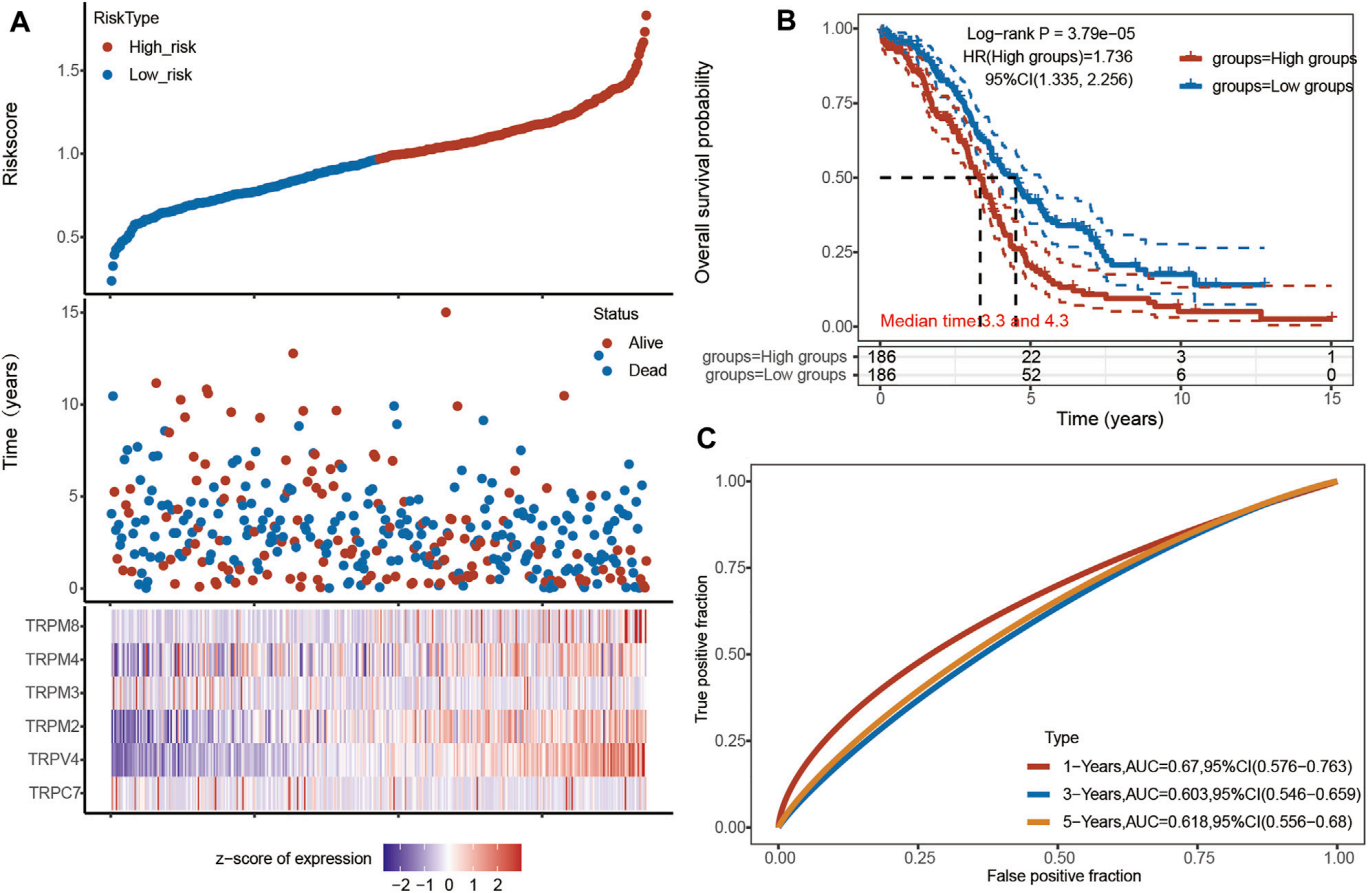
Univariate and multivariate Cox regression analysis of TRPs in OV patients. Annotation: **(A)** The riskscore, survival time and survival status of TCGA Ovarian Serous Cystadenocarcinoma dataset. The top scatterplot represented the riskscore from low (blue) to high risk (red). The scatter plot distribution represented the riskscore of different samples correspond to the survival time and survival status. The heatmap showed the gene expression (*TRPM2, TRPM3, TRPM4, TRPM8*, *TRPV4*, and *TRPC7*) from the signature. **(B)** Kaplan-Meier survival analysis of the risk model, comparison between high and low groups was made by log-rank test. HR represented the hazard ratio of the low-expression samples relative to the high-expression samples. HR> 1 indicated the gene was a risk factor, and HR < 1 indicated the gene was a protective factor. The median survival time (LT50) for different groups was also shown in years. **(C)** ROC curves for verification of the prognostic value of the prognostic model based on six genes [*TRPC7*, *TRPV4*, and *TRPM* (*2,3,4,8*)]. The higher values of AUC corresponded to higher predictive power.

Based on the above research, we focused our attention on the six genes of TRPC7, TRPV4, and TRPM (2,3,4,8). A RS model was constructed using these six genes, and the formula was shown as follows: RS = [TRPC7 expression×−0.031] + [TRPV4 expression × 0.1655] + [TRPM2 expression × 0.0775] + [TRPM3 expression × −0.0467] + [TRPM4 expression × 0.0564] + [TRPM8 expression × 0.1204]. According to this model, we calculated the RS value of each patient and divided the patients into a high-risk group and a low-risk group. It was shown that the high-risk group had a poor prognosis, indicating these six genes had the potential prognostic ability. Cluster analysis showed that TRPV4 performed the best (Figure 6A). The K-M curve showed that OV patients’ OS in the high-risk group was significantly shorter (Figure 6B). The one-year ROC curve was the most obvious among 1-, 3-, and 5-year analyses (Figure 6C). Taken together, TRPV4 provided a promising and rationale druggable target for OV in terms of the overall expression levels of TRPs, the pathological stage of OV, Cox analysis, and prognosis effect.

### Analysis of Gene Alterations, Co-Expression, and Protein/Gene Interactions in the TRP Family of OV Patients

**FIGURE 7:**
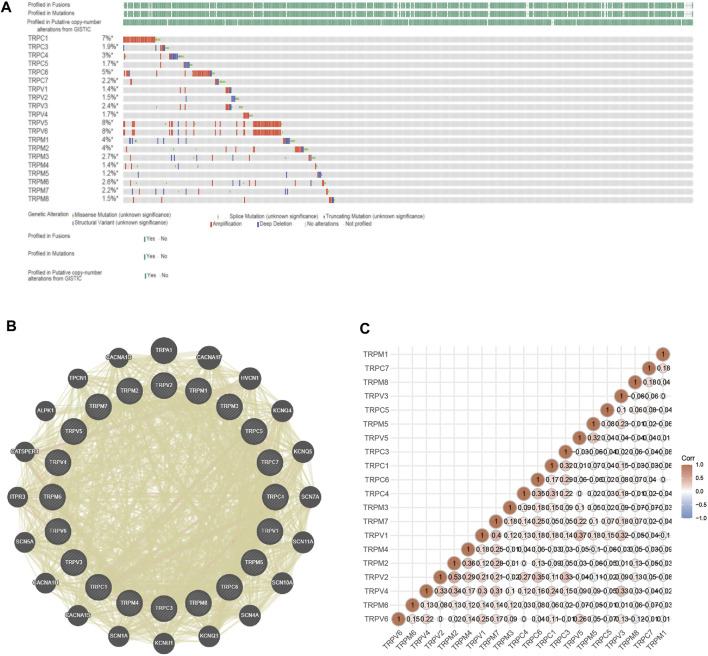
Genetic alterations, adjacent gene network and interaction analysis of TRP family in OV patients. Annotation: **(A)** The mutation status of TRPs in OV samples of TCGA. The mutation probability and mutation types of genes were shown, including missense mutation, splice mutation, truncating mutation, structural variant, amplification and deep deletion. **(B)** PPI network of TRPs. The genes associated with TRPs were shown in the network. **(C)** Protein correlation analysis. The values shown in the intersection of two genes on the axis indicated the correlation. The numbers and the depth of colors (red represented positive correlation and blue represented negative correlation) represented the correlation strength between different genes.

Furthermore, we explored the genetic variation, the function of TRPs, and the correlation between TRP genes. The molecular characteristics of the TRP genes were comprehensively analyzed. First, genetic alterations of the TRP family were analyzed using cBioPortal. As a result, 7% TRPC1, 1.9% TRPC3, 3% TRPC4, 1.7% TRPC5, 5% TRPC6, 2.2% TRPC7, 1.4% TRPV1, 1.5% TRPV2, 2.4% TRPV3, 1.7% TRPV4, and 8% TRPV5, 8%TRPV6, 4% TRPM1, 4% TRPM2, 2.7% TRPM3, 1.4% TRPM4, 1.2% TRPM5, 2.6% TRPM6, 2.2% TRPM7, and 1.5% TRPM8 changed respectively (Figure 7A). Many studies have shown the relationship between TRP mutations and diseases. TRPM6 is very important for Mg^2+^ uptake; TRPM6^P1017R^ mutation impairs channel activity of TRPM6/TRPM7 heteromers, which leads to hypomagnesemia (Chubanov and Gudermann, 2014). K914R mutation of TRPM4 is a Ca^2+^-activated nonselective cation channel linked to human cardiac diseases (Xian et al., 2020). TRPC6^P112Q^ mutation can cause a kidney disease called focal and segmental glomerulosclerosis (FSGS) (Winn et al., 2005). Cantero-Recasens et al. found the loss-of-function TRPV1 variant I585V was associated with a lower risk for childhood asthma (Cantero-Recasens et al., 2010). Mutations for Gly573Ser and Gly573Cys in the TRPV3 gene displayed a hairless or pruritic dermatitis phenotype in rodents (Asakawa et al., 2006). Patients with Spinal Muscular Atrophy have several mutations in TRPV4, namely at R316C and R269H (Verma et al., 2010). GeneMANIA’s results showed that the functions of these TRP genes were mainly related to Ca^2+^ transmembrane transporter activity, ion channel complex, transmembrane transporter complex, transporter complex, and divalent inorganic cation transmembrane transporter activity (Figure 7B). By analyzing the protein interactions, we found that the correlation between TRPV4, TRPV2, and TRPM2 was higher (Figure 7C). We predicted that the higher the correlation of proteins (TRPV4, TRPV2, and TRPM2), the greater the possibility of forming an ion channel tetramer.

### Functional Enrichment Analysis of the TRP Family in OV Patients

**FIGURE 8:**
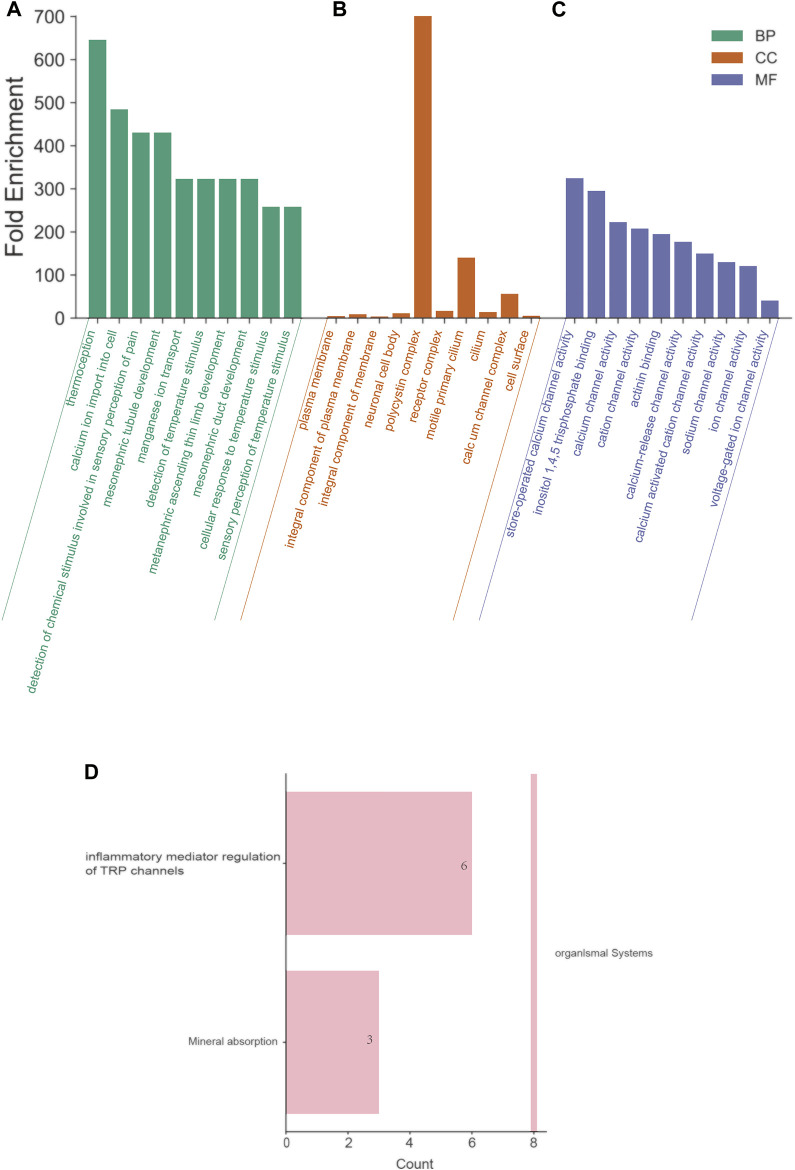
Functional enrichment analysis of the TRP family in OV patients (David6.8). Annotation: GO enrichment analysis and KEGG pathway of TRPs. **(A)** BP: biological processes. **(B)** CC: cell components. **(C)** MF: molecular function. The top ten enriched items were listed in the figure, and the most significant BP, CC and MF were “thermoception”, “polycystin complex”, and “store-operated calcium channel activity”, respectively. **(D)** KEGG pathways. The most significant KEGG pathway was “inflammatory mediator regulation of TRP channel”.

To further explore the functions and the probable mechanism that TRPs may play in OV, GO and KEGG pathway analysis was performed on the TRP family by DAVID6.8, and significant pathways were screened (*p* < 0.05), as shown in Figures 8A–D. In BP (biological process), the most significant enrichment of the top ten is temperature sensation, calcium ion transmembrane transport, detection of chemical stimulation related to pain sensation, tubule development in the middle kidney, manganese ion transport, detection of temperature sensation, upper limb development of the posterior kidney, tubule development in the central kidney, cellular response to temperature stimulation, perception of temperature stimulation. In CC (cell components), plasma membrane, components of the plasma membrane, overall components of the plasma membrane, neuron cell body, polycystic protein complex, receptor complex, active primary cilia, cilia, calcium channel complex, and cell surface are the most significant enrichment items. In MF (molecular function), the considerable enrichment is storage operation calcium channel activity, inositol 1,4,5 triphosphate binding, calcium channel activity, cation channel activity, actinin binding, calcium release channel activity, calcium-activated cation channel activity, sodium channel activity, ion channel activity, and voltage-gated ion channel activity. In KEGG pathway analysis, inflammatory mediator regulation and mineral absorption were significantly enriched in the TRP channel (Figure 8).

### Analysis of TRPs and Immune Cell Infiltration in OV

**FIGURE 9:**
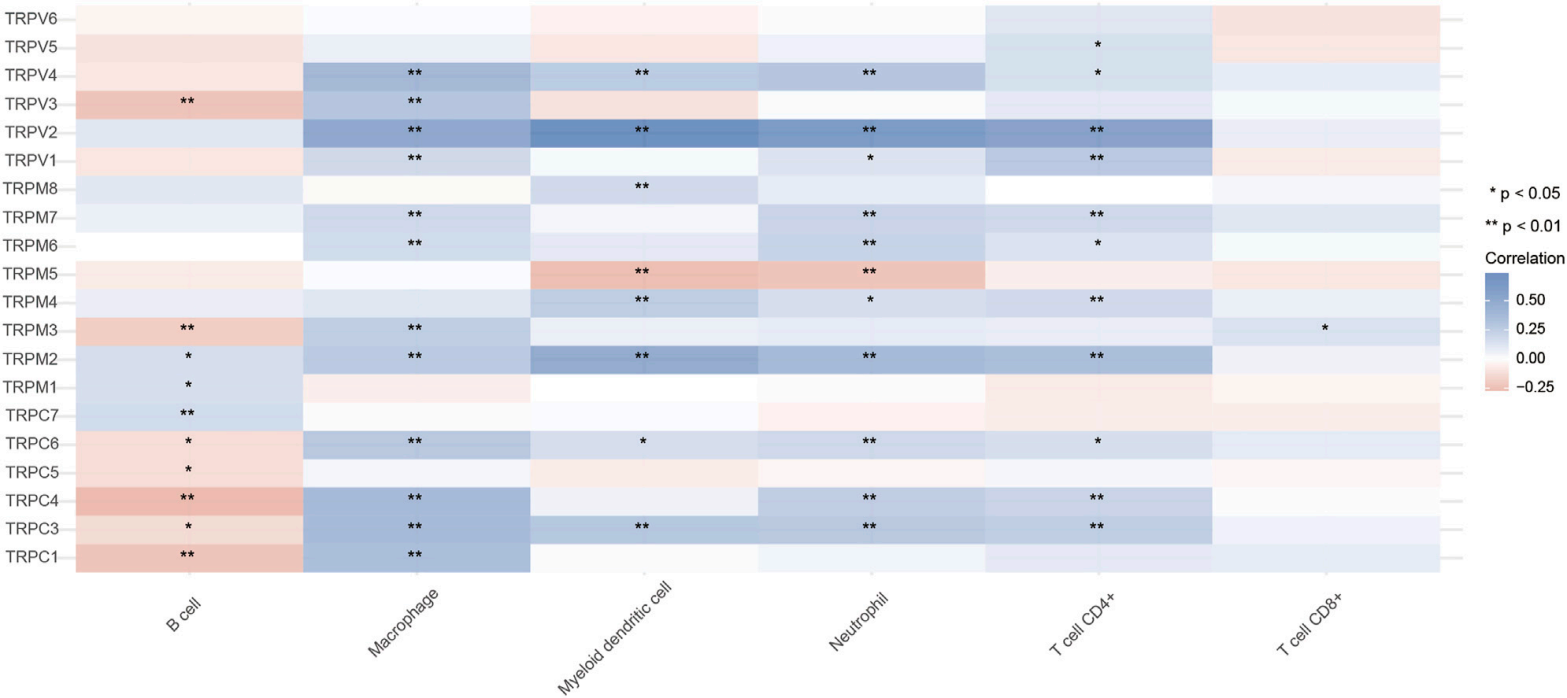
Relationship between TRPs and immune cell infiltration in OV (TIMER). Annotation: Correlation relationships between TRPs and immune cell infiltration. In the diagram, blue represented positive correlation, red represented negative correlation (*p < 0.05, **p < 0.01).

The TRP family is involved in cancer-associated inflammation and immune cell infiltration, thereby influencing the clinical outcome of OV patients. Immune cell infiltration is considered an indicator of the immune microenvironment of OV. To explore the correlation between TRPs and immune response in OV, we systematically analyzed tumor-infiltrating immune/inflammatory cells in OV and evaluated their clinicopathological impact. First, we used TIMER to comprehensively analyze the relationship between the TRP family and immune cell infiltration. As shown in Figure 9, the expression of TRPV4 was significantly correlated with the infiltration of macrophages, myeloid dendritic cells, neutrophils, and CD4^+^ T cells. Cox proportional risk model was applied to analyze the relationship between the TRP family and six tumor-infiltrating cells and the clinical prognosis of OV patients. TRPV2 (*p* = 0.012) and TRPV5 (*p* = 0.003) were significantly correlated with the clinical prognosis of OV patients, while TRPV4 also trended in association with the clinical prognosis of OV patients, although they were not statistically significant (Table 1). The higher levels of tumor-infiltrating macrophages, myeloid dendritic cells, neutrophils, and CD4^+^ T cells with high TRPV4 expression were significantly associated with shorter survival. Tumor-associated macrophages (TAMs) are macrophages that infiltrate tumor tissues and are the most abundant immune cells in the tumor microenvironment. Studies have shown that TAMs could promote the growth and metastasis of tumor cells through multiple pathways (Li et al., 2020). Consistent with this, the TRPV4 was positively related to the macrophage infiltration in the immune microenvironment of OV. Neutrophils are divided into anti-tumor neutrophils and tumor-promoting neutrophils, called N1 and N2, respectively. N2 phenotype of neutrophils secretes molecules such as ROS, arginase, and peroxidase, which inhibit the function of T cells and NK cells and promote tumor growth. In our research, TRPV4 was positively associated with neutrophils, so it was possible that TRPV4 regulated tumor progression by affecting the function of neutrophils. Myeloid dendritic cells with immature phenotypes, abnormal distribution, and dysfunction in the tumor microenvironment are one of the important mechanisms of tumor-induced immune tolerance. TRPV4 may play an important role in regulating the mechanisms of myeloid dendritic cells. The infiltration of many T cells in the tumor microenvironment can also predict the efficacy of immunotherapy. In summary, TRPV4 may be involved in preventing and treating OV by regulating the tumor immune microenvironment. The expression of TRPV4 may be an important indicator of anti-tumor activity.

**TABLE 1:** Cox proportional hazard model of TRPs and six tumor-infiltrating immune cells in OV (TIMER).

	coef	HR	95% CI_l	95%CI_u	p.value	sig
B_cell	−3.157	0.043	0.000	4.961300e+01	0.381	
CD8_Tcell	−3.142	0.043	0.001	2.600000e+00	0.133	
CD4_Tcell	−14.433	0.000	0.000	1.000000e−03	0.000	***
Macrophage	13.373	642,677.104	1,021.396	4.043817e+08	0.000	***
Neutrophil	6.312	551.045	0.035	8.700308e+06	0.201	
Dendritic	0.428	1.534	0.006	3.787210e+02	0.879	
*TRPC1*	0.112	1.118	0.913	1.369000e+00	0.280	
*TRPC5*	0.138	1.148	0.509	2.588000e+00	0.740	
*TRPC6*	0.037	1.038	0.755	1.427000e+00	0.818	
*TRPC7*	−0.334	0.716	0.427	1.201000e+00	0.206	
*TRPV1*	−0.098	0.906	0.538	1.526000e+00	0.711	
*TRPV2*	−0.705	0.494	0.286	8.550000e−01	0.012	*
*TRPV3*	−0.103	0.902	0.477	1.704000e+00	0.750	
*TRPV4*	0.313	1.367	0.950	1.968000e+00	0.092	·
*TRPV5*	−1.063	0.345	0.171	6.980000e−01	0.003	**
*TRPV6*	−0.240	0.787	0.557	1.111000e+00	0.173	
*TRPM1*	0.547	1.727	0.617	4.836000e+00	0.298	
*TRPM3*	−0.176	0.839	0.508	1.386000e+00	0.492	
*TRPM4*	0.018	1.018	0.748	1.386000e+00	0.908	
*TRPM5*	0.231	1.260	0.601	2.641000e+00	0.541	
*TRPM2*	0.509	1.664	0.881	3.145000e+00	0.117	
*TRPM6*	0.515	1.673	0.898	3.115000e+00	0.105	
*TRPM7*	0.167	1.182	0.904	1.546000e+00	0.221	
*TRPM8*	0.047	1.048	0.731	1.504000e+00	0.797	

Notes: *p < 0.05. **p < 0.01. ***p < 0.001.

OV, ovarian serous cystadenocarcinoma HR, hazard ratio; 95% CI_l, 95% confidence interval_lower; 95% CI_u, 95% confidence interval_upper.

### Identification and Establishment of TRPV4 Correlation in OV

**FIGURE 10:**
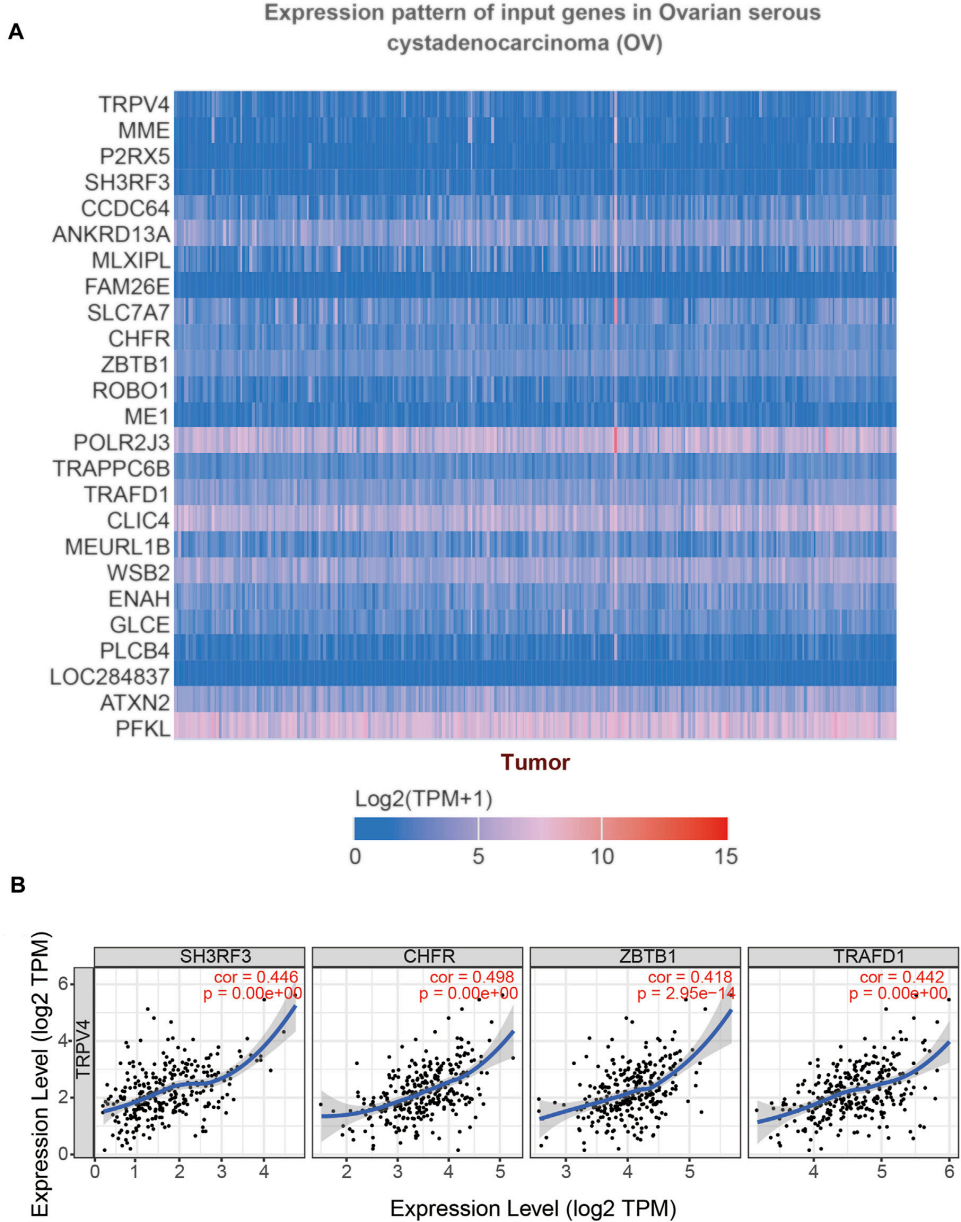
Gene correlation analysis of *TRPV4* in OV (UALCAN). Annotation: **(A)** The top 25 genes related to *TRPV4* were shown in the heatmap. **(B)** Scatter plots illustrated the correlation between *TRPV4* and *SH3RF3*, *CHFR*, *ZBTB1*, *TRAFD1* respectively using the TIMER database.

To understand which TRPV4 related genes ultimately affects the occurrence of OV, we used UALCAN to study the genes related to TRPV4 in OV. The heat map of the top 25 genes showed that genes such as MME, P2RX5, SH3RF3, and CCDC64 were positively related to TRPV4 (Figure 10A), and TIMER was used for verification. The correlation coefficients of SH3RF3 (cor = 0.446), CHFR (cor = 0.498), ZBTB1 (cor = 0.418), and TRAFD1 (cor = 0.442) were high, which may have a good correlation with TRPV4 (Figure 10B). The aberrant expression of SH3RF3 was significantly associated with a higher probability of long-term survival in acute lymphoblastic leukemia (Wang et al., 2015). CHFR was considered a key element for NANOG mediated multi-resistance and stem-like phenotype in immune-edited tumor cells (Woo et al., 2018). ZBTB1, as a transcription factor, prevented DNA damage and p53-mediated apoptosis in replicating immune progenitors, affecting lymphoid development (Grosche et al., 2021). TRAFD1 was reported as a negative feedback regulator that controlled excessive immune responses in vertebrates (Takechi et al., 2016). TNFAIP3 was also proved that it involved in innate immune response (Doostparast Torshizi and Wang, 2017). While genes (SH3RF3, CHFR, ZBTB1, and TRAFD1) prioritized based on TRPV4 positively related genes are significantly up-regulated in the OV, commonly pointing to immune cell function.

## Discussion

Currently, the standard treatment for OV is aggressive surgery and platinum-based chemotherapy. Recurrence and chemotherapy resistance are two major factors leading to high mortality of OV (Li et al., 2016b). Therefore, it is necessary to find biomarkers and the targets for targeted treatment of OV. Our analysis showed that TRPC7, TRPV (4,5,6), and TRPM (2,4,5,8) were up-regulated in OV, while TRPC (1,3,4,6), TRPV (1,2,3), and TRPM (3,7) were down-regulated in OV. According to the prognosis analysis, the high expression of TRPC (5,7), TRPV (1,4), and TRPM (2,4,8) had a poor prognosis, while the low expression of TRPV5, TRPM3 had a poor prognosis. Then, by Cox regression analysis, we found that TRPV4 had a good correlation with the poor prognosis of OV patients, and the up-regulation of TRPV4 was considered to be a cause of OV. The follow-up research showed that TRPV4 might affect the generation of cancer by influencing gene expression or function of SH3RF3, CHFR, ZTB1, and TRAFD1.

At present, there have been many studies on TRPs as a cancer target. The expression of TRPV4 in hepatocellular carcinoma cells is significantly up-regulated, which leads to enhanced EMT and reduced cell apoptosis (Fang et al., 2018). TRPM2 has been proved to promote the growth of prostate cancer cells (Zeng et al., 2010). TRPM4 is up-regulated in breast cancer cells and influences breast cancer by potentially regulating estrogen receptor signal and EMT pathway (Wong and Hussain, 2020). TRPM8 is overexpressed in pancreatic cancer cells and influences on the proliferation and migration ability of pancreatic cancer cells (Yee, 2016). In these studies, the expression levels of TRPV4, TRPM2, TRPM4, and TRPM8 in cancer tissues are significantly increased, which is consistent with our OV research.

Currently, there are some targeted drugs for the OV treatment. Bevacizumab and olaparib, two drugs targeting angiogenesis and DNA damage response pathways, have been approved for the treatment of OV (Grunewald and Ledermann, 2017). Solid tumor growth and progression are reliant on neovascularization (Folkman, 1972). Angiogenesis is complex and regulated by several different endogenous pro-angiogenic and anti-angiogenic factors, such as vascular endothelial growth factors (Schmid and Oehler, 2015). Bevacizumab acts on vascular endothelial growth factors to treat OV (Burger et al., 2011). Actually, Olaparib is a poly ADP ribose polymerase inhibitor to effectively inhibit the body from automatically repairing the existing DNA damage.

Also, there are already some drugs that target TRPs to treat related cancers. For example, GSK2193874 is a TRPV4 blocker with an oral activity which inhibits the formation of the abdominal aortic aneurysm by reducing the activation of smooth muscle cells and the migration of neutrophils across cells (Shannon et al., 2020). In prostate cancer, D-3263 (an orally bioavailable small molecule that targets TRPM8) binds and activates TRPM8, induces Ca^2+^ to enter cells, causing the destruction of calcium ion homeostasis and cell death of tumor cells expressing TRPM8. At the same time, D-3263 can reduce the level of dihydro-testosterone to inhibit prostate cancer (Santoni and Farfariello, 2011).

In the review of this study, there were limitations in analyzing data using only bioinformatic methods, and the design of experimental methods was relatively simple. This study pointed out the TRP family may influence the generation and development of OV, especially TRPV4. The physiological functions of TRPV4 are thermosensation, mechanosensation, osmosensation, nociception, endothelium vasomotor control and shear stress sensor, modulation of cell migration, and control of adherens junctions in the skin (Yu et al., 2019). These functions, especially cell migration and adhesion, may be related to the poor prognosis of OV. However, few studies were on whether TRPV4 could be used as a therapeutic target for OV. Cheng et al. conducted Cox analysis and K-M survival analysis using TCGA’s pan-cancer dataset and proved that TRPV4 could be used as a prognostic marker of ovarian cancer (Wang et al., 2021). In our study, we analyzed the expression pattern, survival, and prognosis of the TRP family in OV patients, and also concluded that TRPV4 could be used as a prognostic marker and therapy target for OV. This further verified the great potential of TRPV4 in ovarian cancer. Still, we looked forward to further studies on the molecular mechanism of TRPs as a biomarker and therapeutic target for OV to provide a more valuable reference for targeted treatment for OV.

## Data Availability

The original contributions presented in the study are included in the article/Supplementary Material; further inquiries can be directed to the corresponding authors.

## Abbreviations

AUC, area under the curve; BP, biological process; BTB1, zinc finger and btb domain-containing protein 1; CC, cell component; COL11A1, collagen type XI alpha 1 chain; CCDC64, coiled-coil domain containing 64 proteins; CHFR, an e3 ligase for histone deacetylase 1 protein; EMT, epithelial–mesenchymal transition; FSGS, focal and segmental glomerulosclerosis; K-M, kaplan-meier; MF, molecular function; MME, membrane metalloendopeptidase; NANOG, one of the key regulators in maintaining the tumor-initiating stem-like cells; OV, ovarian serous cystadenocarcinoma; OS, overall survival; PPI, protein-protein interaction; POSTN, periostin; P2RX5, purinergic receptor P2X5; RFS, recurrence-free survival; RS, risk score; ROC, receiver operating characteristic; SH3RF3, src homology 3 domain containing ring finger 3; TAMs, tumor-associated macrophages; TCGA, the cancer genome atlas; TRP, transient receptor potential; TRPC, transient receptor potential canonical; TRPV, transient receptor potential vanilloid; TRPM, ransient receptor potential melastatin; TRAFD1, a tumor necrosis factor receptor-associated factor-type zinc finger domain containing protein 1; TNFAIP3, the tumor necrosis factor alpha-induced protein 3.

## References

[B1] Asakawa M. Yoshioka T. Matsutani T. Hikita I. Suzuki M. Oshima I. (2006). Association of a Mutation in TRPV3 with Defective Hair Growth in Rodents. J. Investigative Dermatology 126 (12), 2664–2672. 10.1038/sj.jid.5700468 16858425

[B2] Burger R. A. Brady M. F. Bookman M. A. Fleming G. F. Monk B. J. Huang H. (2011). Incorporation of Bevacizumab in the Primary Treatment of Ovarian Cancer. N. Engl. J. Med. 365 (26), 2473–2483. 10.1056/nejmoa1104390 22204724

[B3] Cantero-Recasens G. Gonzalez J. R. Fandos C. Duran-Tauleria E. Smit L. A. M. Kauffmann F. (2010). Loss of Function of Transient Receptor Potential Vanilloid 1 (TRPV1) Genetic Variant Is Associated with Lower Risk of Active Childhood Asthma. J. Biol. Chem. 285 (36), 27532–27535. 10.1074/jbc.c110.159491 20639579 PMC2934619

[B4] Cerami E. Gao J. Dogrusoz U. Gross B. E. Sumer S. O. Aksoy B. A. (2012). The cBio Cancer Genomics Portal: An Open Platform for Exploring Multidimensional Cancer Genomics Data: Figure 1. Cancer Discov. 2 (5), 401–404. 10.1158/2159-8290.cd-12-0095 22588877 PMC3956037

[B5] Chandrashekar D. S. Bashel B. Balasubramanya S. A. H. Creighton C. J. Ponce-Rodriguez I. Chakravarthi B. V. S. K. (2017). UALCAN: A Portal for Facilitating Tumor Subgroup Gene Expression and Survival Analyses. Neoplasia 19 (8), 649–658. 10.1016/j.neo.2017.05.002 28732212 PMC5516091

[B6] Chen X. Sooch G. Demaree I. S. White F. A. Obukhov A. G. (2020). Transient Receptor Potential Canonical (TRPC) Channels: Then and Now. Cells 9 (9), 1983. 10.3390/cells9091983 PMC7565274 32872338

[B7] Chubanov V. Gudermann T. (2014). Trpm6. Handb. Exp. Pharmacol. 222, 503–520. 10.1007/978-3-642-54215-2_20 24756719

[B8] Doostparast Torshizi A. Wang K. (2017). Deconvolution of Transcriptional Networks in Post-Traumatic Stress Disorder Uncovers Master Regulators Driving Innate Immune System Function. Sci. Rep. 7 (1), 14486. 10.1038/s41598-017-15221-y 29101382 PMC5670244

[B9] Fang Y. Liu G. Xie C. Qian K. Lei X. Liu Q. (2018). Pharmacological Inhibition of TRPV4 Channel Suppresses Malignant Biological Behavior of Hepatocellular Carcinoma via Modulation of ERK Signaling Pathway. Biomed. Pharmacother. 101, 910–919. 10.1016/j.biopha.2018.03.014 29635900

[B10] Folkman J. (1972). Anti-Angiogenesis. Ann. Surg. 175 (3), 409–416. 10.1097/00000658-197203000-00014 5077799 PMC1355186

[B11] Gao Y. Liao P. (2019). TRPM4 Channel and Cancer. Cancer Lett. 454, 66–69. 10.1016/j.canlet.2019.04.012 30980865

[B12] Grosche S. Marenholz I. Esparza-Gordillo J. Arnau-Soler A. Pairo-Castineira E. Rüschendorf F. (2021). Rare Variant Analysis in Eczema Identifies Exonic Variants in DUSP1, NOTCH4 and SLC9A4. Nat. Commun. 12 (1), 6618. 10.1038/s41467-021-26783-x 34785669 PMC8595373

[B13] Grunewald T. Ledermann J. A. (2017). Targeted Therapies for Ovarian Cancer. Best Pract. Res. Clin. Obstetrics Gynaecol. 41, 139–152. 10.1016/j.bpobgyn.2016.12.001 28111228

[B14] Hao H. B. Webb S. E. Yue J. Moreau M. Leclerc C. Miller A. L. (2018). TRPC3 Is Required for the Survival, Pluripotency and Neural Differentiation of Mouse Embryonic Stem Cells (mESCs). Sci. China Life Sci. 61 (3), 253–265. 10.1007/s11427-017-9222-9 29392682

[B15] Huang D. W. Sherman B. T. Lempicki R. A. (2009). Bioinformatics Enrichment Tools: Paths toward the Comprehensive Functional Analysis of Large Gene Lists. Nucleic Acids Res. 37 (1), 1–13. 10.1093/nar/gkn923 19033363 PMC2615629

[B16] Huang D. W. Sherman B. T. Lempicki R. A. (2009). Systematic and Integrative Analysis of Large Gene Lists Using DAVID Bioinformatics Resources. Nat. Protoc. 4 (1), 44–57. 10.1038/nprot.2008.211 19131956

[B17] Huang Y. Fliegert R. Guse A. H. Lü W. Du J. (2020). A Structural Overview of the Ion Channels of the TRPM Family. Cell Calcium 85, 102111. 10.1016/j.ceca.2019.102111 31812825 PMC7050466

[B18] Hutter C. Zenklusen J. C. (2018). The Cancer Genome Atlas: Creating Lasting Value beyond its Data. Cell 173 (2), 283–285. 10.1016/j.cell.2018.03.042 29625045

[B19] Kaneko Y. Szallasi A. (2014). Transient Receptor Potential (TRP) Channels: a Clinical Perspective. Br. J. Pharmacol. 171 (10), 2474–2507. 10.1111/bph.12414 24102319 PMC4008995

[B20] Lee Y. T. Tan Y. J. Oon C. E. (2018). Molecular Targeted Therapy: Treating Cancer with Specificity. Eur. J. Pharmacol. 834, 188–196. 10.1016/j.ejphar.2018.07.034 30031797

[B21] Li B. Severson E. Pignon J.-C. Zhao H. Li T. Novak J. (2016). Comprehensive Analyses of Tumor Immunity: Implications for Cancer Immunotherapy. Genome Biol. 17 (1), 174. 10.1186/s13059-016-1028-7 27549193 PMC4993001

[B22] Li H.-S. Xu X.-Z. S. Montell C. (1999). Activation of a TRPC3-dependent Cation Current through the Neurotrophin BDNF. Neuron 24 (1), 261–273. 10.1016/s0896-6273(00)80838-7 10677043

[B23] Li S. Cao J. Zhang W. Zhang F. Ni G. Luo Q. (2016). Protein Tyrosine Phosphatase PTPN3 Promotes Drug Resistance and Stem Cell-like Characteristics in Ovarian Cancer. Sci. Rep. 6, 36873. 10.1038/srep36873 27833130 PMC5105059

[B24] Li T. Fan J. Wang B. Traugh N. Chen Q. Liu J. S. (2017). TIMER: A Web Server for Comprehensive Analysis of Tumor-Infiltrating Immune Cells. Cancer Res. 77 (21), e108–e110. 10.1158/0008-5472.can-17-0307 29092952 PMC6042652

[B25] Li Y. Hodge J. Liu Q. Wang J. Wang Y. Evans T. D. (2020). TFEB Is a Master Regulator of Tumor-Associated Macrophages in Breast Cancer. J. Immunother. Cancer 8 (1), 543. 10.1136/jitc-2020-000543 PMC7269543 32487570

[B26] Liu H. Wang A. Ma Y. (2020). Increased Expression of LYNX1 in Ovarian Serous Cystadenocarcinoma Predicts Poor Prognosis. Biomed. Res. Int. 2020, 1392674. 10.1155/2020/1392674 33299855 PMC7710416

[B27] Moran M. M. (2018). TRP Channels as Potential Drug Targets. Annu. Rev. Pharmacol. Toxicol. 58, 309–330. 10.1146/annurev-pharmtox-010617-052832 28945977

[B28] Pan Z. Yang H. Reinach P. S. (2011). Transient Receptor Potential (TRP) Gene Superfamily Encoding Cation Channels. Hum. Genomics 5 (2), 108–116. 10.1186/1479-7364-5-2-108 21296744 PMC3525231

[B29] Poteser M. Graziani A. Eder P. Yates A. Mächler H. Romanin C. (2008). Identification of a Rare Subset of Adipose Tissue-Resident Progenitor Cells, Which Express CD133 and TRPC3 as a VEGF-Regulated Ca2+entry Channel. FEBS Lett. 582 (18), 2696–2702. 10.1016/j.febslet.2008.06.049 18602918

[B31] Santoni G. Farfariello V. Amantini C. (2011). TRPV Channels in Tumor Growth and Progression. Adv. Exp. Med. Biol. 704, 947–967. 10.1007/978-94-007-0265-3_49 21290335

[B32] Santoni G. Farfariello V. (2011). TRP Channels and Cancer: New Targets for Diagnosis and Chemotherapy. Emiddt 11 (1), 54–67. 10.2174/187153011794982068 21348820

[B33] Schmid B. C. Oehler M. K. (2015). Improvements in Progression-free and Overall Survival Due to the Use of Anti-angiogenic Agents in Gynecologic Cancers. Curr. Treat. Options Oncol. 16 (1), 318. 10.1007/s11864-014-0318-0 25750175

[B34] Shannon A. H. Elder C. T. Lu G. Su G. Mast A. Salmon M. D. (2020). Pharmacologic Inhibition of Transient Receptor Channel Vanilloid 4 Attenuates Abdominal Aortic Aneurysm Formation. FASEB J. 34 (7), 9787–9801. 10.1096/fj.202000251r 32506673 PMC8162061

[B35] Shi Z. Zhao Q. Lv B. Qu X. Han X. Wang H. (2021). Identification of Biomarkers Complementary to Homologous Recombination Deficiency for Improving the Clinical Outcome of Ovarian Serous Cystadenocarcinoma. Clin. Transl. Med. 11 (5), e399. 10.1002/ctm2.399 34047476 PMC8131501

[B36] Siveen K. S. (2020). TRPV2: A Cancer Biomarker and Potential Therapeutic Target. Dis. Markers 2020, 8892312. 10.1155/2020/8892312 33376561 PMC7746447

[B30] Stewart C. Ralyea C. Lockwood S. (2019). Ovarian Cancer: An Integrated Review. Semin. Oncol. Nurs. 35 (2), 151–156. 10.1016/j.soncn.2019.02.001 30867104

[B37] Takechi R. Galay R. L. Matsuo T. Maeda H. Kusakisako K. Talactac M. R. (2016). Role of the Tumor Necrosis Factor Receptor-Associated Factor-type Zinc Finger Domain Containing Protein 1 (TRAFD1) from the Hard Tick Haemaphysalis Longicornis in Immunity against Bacterial Infection. Ticks Tick-borne Dis. 7 (1), 36–45. 10.1016/j.ttbdis.2015.08.002 26283173

[B38] Tang Z. Li C. Kang B. Gao G. Li C. Zhang Z. (2017). GEPIA: a Web Server for Cancer and Normal Gene Expression Profiling and Interactive Analyses. Nucleic Acids Res. 45 (W1), W98–W102. 10.1093/nar/gkx247 28407145 PMC5570223

[B39] Tiapko O. Groschner K. (2018). TRPC3 as a Target of Novel Therapeutic Interventions. Cells 7 (7), 83. 10.3390/cells7070083 PMC6071100 30037143

[B40] Verma P. Kumar A. Goswami C. (2010). TRPV4-mediated Channelopathies. Channels 4 (4), 319–328. 10.4161/chan.4.4.12905 20676052

[B41] Wang J. Mi J.-Q. Debernardi A. Vitte A.-L. Emadali A. Meyer J. A. (2015). A Six Gene Expression Signature Defines Aggressive Subtypes and Predicts Outcome in Childhood and Adult Acute Lymphoblastic Leukemia. Oncotarget 6 (18), 16527–16542. 10.18632/oncotarget.4113 26001296 PMC4599287

[B42] Wang K. Feng X. Zheng L. Chai Z. Yu J. You X. (2021). TRPV4 Is a Prognostic Biomarker that Correlates with the Immunosuppressive Microenvironment and Chemoresistance of Anti-cancer Drugs. Front. Mol. Biosci. 8, 690500. 10.3389/fmolb.2021.690500 34262942 PMC8273915

[B43] Webb P. M. Jordan S. J. (2017). Epidemiology of Epithelial Ovarian Cancer. Best Pract. Res. Clin. Obstetrics Gynaecol. 41, 3–14. 10.1016/j.bpobgyn.2016.08.006 27743768

[B44] Winn M. P. Conlon P. J. Lynn K. L. Farrington M. K. Creazzo T. Hawkins A. F. (2005). A Mutation in the TRPC6 Cation Channel Causes Familial Focal Segmental Glomerulosclerosis. Science 308 (5729), 1801–1804. 10.1126/science.1106215 15879175

[B45] Wong K. K. Hussain F. A. (2020). TRPM4 Is Overexpressed in Breast Cancer Associated with Estrogen Response and Epithelial-Mesenchymal Transition Gene Sets. PLoS One 15 (6), e0233884. 10.1371/journal.pone.0233884 32484822 PMC7266295

[B46] Woo S. R. Lee H.-J. Oh S. J. Kim S. Park S.-H. Lee J. (2018). Stabilization of HDAC1 via TCL1-pAKT-CHFR axis Is a Key Element for NANOG-Mediated Multi-Resistance and Stem-like Phenotype in Immune-Edited Tumor Cells. Biochem. Biophysical Res. Commun. 503 (3), 1812–1818. 10.1016/j.bbrc.2018.07.118 30060952

[B47] Wu Y.-H. Chang T.-H. Huang Y.-F. Huang H.-D. Chou C.-Y. (2014). COL11A1 Promotes Tumor Progression and Predicts Poor Clinical Outcome in Ovarian Cancer. Oncogene 33 (26), 3432–3440. 10.1038/onc.2013.307 23934190

[B48] Wu Y.-H. Huang Y.-F. Chang T.-H. Chen C.-C. Wu P.-Y. Huang S.-C. (2021). COL11A1 Activates Cancer-Associated Fibroblasts by Modulating TGF-Β3 through the NF-Κb/igfbp2 axis in Ovarian Cancer Cells. Oncogene 40 (26), 4503–4519. 10.1038/s41388-021-01865-8 34117361

[B49] Xian W. Wang H. Moretti A. Laugwitz K. L. Flockerzi V. Lipp P. (2020). Domain Zipping and Unzipping Modulates TRPM4's Properties in Human Cardiac Conduction Disease. FASEB J. 34 (9), 12114–12126. 10.1096/fj.202000097rr 32681584

[B50] Yang D. Kim J. (2020). Emerging Role of Transient Receptor Potential (TRP) Channels in Cancer Progression. BMB Rep. 53 (3), 125–132. 10.5483/bmbrep.2020.53.3.016 32172727 PMC7118349

[B51] Yee N. S. (2016). TRPM8 Ion Channels as Potential Cancer Biomarker and Target in Pancreatic Cancer. Adv. Protein Chem. Struct. Biol. 104, 127–155. 10.1016/bs.apcsb.2016.01.001 27038374

[B52] Yu S. Huang S. Ding Y. Wang W. Wang A. Lu Y. (2019). Transient Receptor Potential Ion-Channel Subfamily V Member 4: a Potential Target for Cancer Treatment. Cell Death Dis. 10 (7), 497. 10.1038/s41419-019-1708-9 31235786 PMC6591233

[B53] Yue H. Li W. Chen R. Wang J. Lu X. Li J. (2021). Stromal POSTN Induced by TGF-Β1 Facilitates the Migration and Invasion of Ovarian Cancer. Gynecol. Oncol. 160 (2), 530–538. 10.1016/j.ygyno.2020.11.026 33317907

[B54] Zeng X. Sikka S. C. Huang L. Sun C. Xu C. Jia D. (2010). Novel Role for the Transient Receptor Potential Channel TRPM2 in Prostate Cancer Cell Proliferation. Prostate Cancer Prostatic Dis. 13 (2), 195–201. 10.1038/pcan.2009.55 20029400 PMC2871075

[B55] Zhang Y. Lun X. Guo W. (2020). Expression of TRPC1 and SBEM Protein in Breast Cancer Tissue and its Relationship with Clinicopathological Features and Prognosis of Patients. Oncol. Lett. 20 (6), 392. 10.3892/ol.2020.12255 33193852 PMC7656111

